# Lithium Suppresses Hedgehog Signaling via Promoting ITCH E3 Ligase Activity and Gli1–SUFU Interaction in PDA Cells

**DOI:** 10.3389/fphar.2017.00820

**Published:** 2017-11-16

**Authors:** Xinshuo Wang, Zijian Fang, Anlin Wang, Cheng Luo, Xiaodong Cheng, Meiling Lu

**Affiliations:** ^1^State Key Laboratory of Natural Medicines, School of Life Sciences and Technology, China Pharmaceutical University, Nanjing, China; ^2^School of Medicine and Life Sciences, Nanjing University of Chinese Medicine, Nanjing, China; ^3^Department of Integrative Biology and Pharmacology, Texas Therapeutics Institute, The Brown Foundation Institute of Molecular Medicine, University of Texas Health Science Center at Houston, Houston, TX, United States

**Keywords:** Gli1, lithium, ITCH, GSK3β, pancreatic cancer, ubiquitination, hedgehog signaling

## Abstract

Dysregulation of Hedgehog (Hh) signaling pathway is one of the hallmarks of pancreatic ductal adenocarcinoma (PDA). Lithium, a clinical mood stabilizer for the treatment of mental disorders, is known to suppress tumorigenic potential of PDA cells by targeting the Hh/Gli signaling pathway. In this study, we investigated the molecular mechanism of lithium induced down-regulation of Hh/Gli1. Our data show that lithium promotes the poly-ubiquitination and proteasome-mediated degradation of Gli1 through activating E3 ligase ITCH. Additionally, lithium enhances interaction between Gli1 and SUFU via suppressing GSK3β, which phosphorylates SUFU and destabilizes the SUFU-Gli1 inhibitory complex. Our studies illustrate a novel mechanism by which lithium suppresses Hh signaling via simultaneously promoting ITCH-dependent Gli1 ubiquitination/degradation and SUFU-mediated Gli1 inhibition.

## Introduction

Pancreatic cancer currently is the third leading cause cancer-related death in the United States and will likely surpass colorectal cancer in term of mortality by 2020. In contrast to the steady increase in survival for most cancers, advances have been slow for pancreatic ductal adenocarcinoma (PDA), for which the 5-year relative survival is currently 8% with little improvement over the past 30 years (Siegel et al., 2017). Therefore, a better understanding of PDA pathphysiology and the development of novel therapeutic options are urgently needed.

Hedgehog (Hh)/Gli signaling is a key pathway critical for embryonic development and tissue homeostasis. Aberrant activation of Hh/Gli signaling has been linked to several types of cancer, including those of the skin, brain, lungs, prostate, gastrointestinal tract, blood, and colon (Karhadkar et al., 2004; Clement et al., 2007; Stecca et al., 2007; Yuan et al., 2007; Xie, 2008; Varnat et al., 2009; Takezaki et al., 2011; Raz et al., 2012; Santini et al., 2012; Wang et al., 2012; Gonnissen et al., 2013; Pandolfi and Stecca, 2015). Hh signaling also plays a critical role in the initiation and development of PDA (Thayer et al., 2003; Morris et al., 2010). In mammals, Hh signaling is mainly transduced by three members of Gli transcriptional regulators, Gli1, Gli2, and Gli3. Each of them plays different function and possesses unique properties in gene expression and regulation. Unlike Gli2 or Gli3, Gli1 is primarily a transcriptional activator and is not processed to a repressor form (Lee et al., 1997). Gli1 can be degraded by the proteasome via two different ubiquitin-dependent processing pathways mediated by β-TrCP (β-transducin repeat-containing protein) and ITCH, two distinct E3 ubiquitin ligases known to recognize and inactivate Gli1 in the cytosol (Lee et al., 1997; Di Marcotullio et al., 2006; Huntzicker et al., 2006). It has also been reported that PCAF (p53-mediated elevation of the acetyltransferase p300/CBP-associated factor) can act as a special ubiquitin E3 ligase of Gli1 and could inhibit Hh/Gli1 signaling in p53-dependent response to genotoxic stress (Mazzà et al., 2013). In addition to ubiquitin-mediated degradation, Hh pathway can also be regulated by suppressor of fused homolog (SUFU), which exerts its inhibitory effects by interacting with Gli1 and sequestering it in the cytosol (Ding et al., 1999; Kogerman et al., 1999; Stone et al., 1999; Dunaeva et al., 2003). As a consequence, Gli1 proteins are either degraded or tightly confined in cytoplasm under basal conditions whereas the activation of Hh/Gli signaling pathway is associated with the high Gli1 level in nucleus where it exerts strong mitogenic and prosurvival activities. Thus, down-regulation of Hh/Gli signaling pathway by targeting Gli1 protein nuclear accumulation may represent a new avenue for tumorigenesis suppression and antitumor therapy (Lauth et al., 2007).

Lithium is widely used as an efficacious treatment of bipolar disorder and acute mania (Schou et al., 1954; Bowden et al., 1994; Geddes and Miklowitz, 2013). While a precise mechanism is still lacking, the therapeutic effects of lithium are typically attributed to its inhibitory activity toward GSK3β, which cross-talks with multiple key signaling transduce pathways (Klein and Melton, 1996; O’Brien and Klein, 2009). In patients with bipolar disorder, lithium treatment is associated with a dose-dependent reduction of overall cancer risk, particularly in patients with respiratory, gastrointestinal, and endocrine cancers (Huang et al., 2016; Martinsson et al., 2016). In recent years, extensive studies have been carried out to explore the anti-tumor activity of lithium in various cancers such as neuroglioma (Freland and Beaulieu, 2012), colon cancer (Vidal et al., 2011), medulloblastoma (Ronchi et al., 2011), and hepatocellular carcinoma (Erdal et al., 2005). Other regulation functions of lithium have also been reported, such as its influence on the circadian clock mechanism through targeting E3 ligases Arf-bp1 and Pam (Yin et al., 2010). Our recent study indicates that lithium inhibits pancreatic cancer cell proliferation through modulating the Hh/Gli signaling pathway by suppressing Gli1 (Peng et al., 2013). However, the mechanisms of Li-mediated Gli1 down-regulation remain unidentified.

In this study, we report that lithium inhibits Hh/Gli1 signaling through two concerted pathways. On the one hand, lithium accelerates the degradation of Gli1 through promoting ITCH-mediated ubiquitination. On the other hand, lithium prevents the dissociation of SUFU/Gli1 complex, thus the translocation of Gli1 to nucleus by inhibiting GSK3β.

## Materials and Methods

### Cell Line and Reagents

Human pancreatic cancer cell lines PANC-1 and human embryonic kidney 293 (HEK293) cells were obtained from the Cell Bank of Type Culture Collection of Chinese Academy of Sciences (Shanghai, China) and cultured in Dulbecco’s modified eagle medium (DMEM) containing 10% fetal bovine serum (FBS) in a humidified 5% CO_2_ incubator at 37°C. LiCl (CAS NO. 7447-41-8) was purchased from EMD chemicals (EMD, Germany). MG132 (CAS NO. 133407-82-6) and PYR-41 (CAS NO. 418805-02-4) were purchased from Selleck (Selleck, United States). Monoclonal antibodies against Gli1, ITCH, β-TrCP, PCAF, HA, Myc-tag, GSK3β, p-GSK3β-Ser9, p-GSK3β-Tyr216, SUFU, GAPDH, and β-actin were obtained from CST (Cell Signaling Technology, United States). Other common regents were purchased from Sigma (Sigma–Aldrich, United States).

### Plasmids and Transfections

Full length human Gli1 was subcloned into pCDNA3.1 (+) with a tandem myc- and his-tag. The truncated Gli1 [Myc-ΔGli1 (1–300 AA) and ΔGli1-His (755–1106 AA)] were constructed into pCDNA3.1 (+) with a myc- and his-tag, respectively. pCDNA3.1 (+)-ITCH was purchased from Generay Biotech, Co., Ltd. (Generay, China). Eight Ub repeats was subcloned into pCDNA3.1 (+) with an HA tag. Gli1 mutations (K929A, K960A, and K1003A) were constructed by using a Ligation-Free Cloning System mutagenesis kit (abm, China). Individual constructions were transfected into PANC-1 cells using Lipofectamine^®^ 3000 reagent (Invitrogen, United States) according to the manufacturer’s instructions.

### Quantitative RT-PCR

Total RNA was isolated using RNaEX^TM^ Total RNA Isolation Solution (Generay, China) according to the manufacturer’s protocol. cDNA was prepared using HiScript^®^ Q RT SuperMix (Vazyme, China). Quantitative PCR was performed using AceQ^®^ Green I (Vazyme, China) with a Roche Applied Science LightCyclerTM 480 (Roche, Swiss). The primers were as follows: ITCH (ITCH F, TTCGTGTGTGGAGTCACCAG; ITCH R, TGTCACCTCC AAGCTGCAAA). The relative mRNA level of ITCH was calculated by 2^-ΔΔC_T_^ method and normalized with GAPDH as endogenous reference gene.

### Immunoblotting

For immunoblotting, cells treated with different compounds were collected at the indicated time and lysed with the RIPA lysis buffer (Beyotime, China). Quantification of the cell lysates were assayed with a BCA protein assay kit (Generay, China). 20 μg proteins were subjected to electrophoresis using 12% SDS-PAGE gels and transferred onto polyacrylamide difluoride (PVDF) membranes (Millipore, United States). After being blocked with 5% bovine serum albumin (BSA) in Tris-buffered saline with Tween-20, membranes were incubated with individual primary antibodies (1:1000) at 4°C overnight followed by secondary antibody (1:5000) at room temperature for 1 h. The blots were detected by ECL Western blotting Detection System (Millipore, United States). The band density was measured by LabWorks Image Acquisition and Analysis Software (UVP, Upland, CA, United States) and normalized with band density of β-actin or GAPDH.

### Co-immunoprecipitation

Co-immunoprecipitation was performed using Protein A/G plus Argrose Beads (Cell Signaling Technology, United States) according to the manufacture’s instructions. For Gli1 and SUFU co-immunoprecipitation, 5 μg of Gli1 and SUFU antibodies was used per sample, respectively. Samples-antibodies complex were incubated with the beads at 4°C for 4 h. Sample-antibody-beads complex were eluted with lysis buffer before analysis with immunoblotting.

### *In Vitro* Ubiquitylation Assay

PANC-1 cells were transfected with pCDNA3.1 (+)-ubiquitin along with pCDNA3.1 (+)-Gli1 or pCDNA3.1 (+)-ITCH. At 36 h after transfection, cells were treated with 10 mM LiCl and 2 μM MG132 for 24 h and lysed in immunoprecipitation buffer (Beyotime, China). About 1 mg of protein lysates were used in immunoprecipitation assays with PureProteome^TM^ NHS FlexiBind Magnetic Bead Kit (Millipore, United States) according to the manufacturer’s instructions. Briefly, the PureProteome^TM^ NHS FlexiBind Magnetic Beads coupled with the specific antibodies were prepared and cell lysates were then mixed with the beads. After incubation for 2 h at room temperature, captured immuno-complex were separated by 4–20% SDS-PAGE gradient gels (Bio-Rad, United States) and immunoblotting was performed with anti-HA or anti-Myc antibody. 5 μL of IP eluent was used for immunoblotting analysis to evaluate the expression of ubiquitin-HA and Gli1.

### Immunofluorescence

Immunofluorescence staining was performed using Gli1 immunofluorescence antibody (Cell Signaling Technology, United States). Briefly, PANC-1 cells were treated with 2 μM PYR-41 only or co-treated with 2 μM PYR and 10 mM LiCl or 10 μM CHIR99021 for 24 h. The cells were then fixed by 4% formaldehyde diluted in 1× PBS. After blocked with blocking buffer for 60 min, cells were incubated with Gli1 immunofluorescence antibody at 4°C for 1 h. After washing with PBS, cells were incubated with fluorochrome-conjugated secondary antibody (ThermoFisher Scientific, United States) (1:50) at room temperature for 30 min. For nucleus staining, DAPI was added to the cells. Images were captured using Olympus CX 31 microscope and analyzed by Image J software.

### Statistics Analysis

The results were presented as the mean ± SEM. Statistics significance of the experiments between the different groups was analyzed by one-way analysis of variance followed by the Student-Newman-Keuls comparisons. *P* < 0.05 was considered to be statistics significant.

## Results

### Lithium Reduces Cellular Gli1 Levels by Upregulating ITCH

**FIGURE 1:**
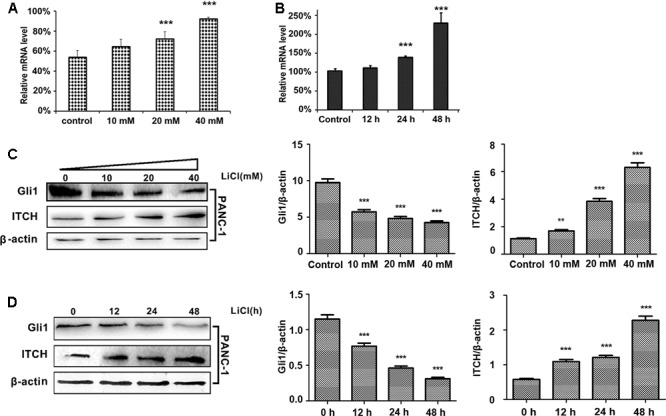
Lithium induces the up-regulation of ITCH level and the down-regulation of Gli1. **(A)** The relative mRNA level of ITCH in PANC-1 cells was up-regulated in a dose-dependent manner by lithium treatment for 24 h. **(B)** The relative mRNA level of ITCH in PANC-1 cells was up-regulated in a time-dependent manner when treated by 10 mM LiCl. **(C)** The Gli1 protein level was down-regulated accompanied by the up-regulation of ITCH in a dose-dependent manner when using lithium to treat PANC-1 cells for 24 h. Histogram represented summarized results from three independent experiments. **(D)** The Gli1 protein level was down-regulated accompanied by the up-regulation of ITCH in a time-dependent manner by the treatment of 10 mM lithium. Histogram represented summarized results from three independent experiments. ^∗∗^*P* < 0.01 and ^∗∗∗^*P* < 0.005.

To test the hypothesis that lithium may suppress Gli1 protein stability by via ubiquitination mediated degradation, we investigate the effect of lithium on the expression of E3 ligase ITCH. PANC-1 cells were treated with different concentrations of lithium chloride (10, 20, and 40 mM) for 12, 24, and 48 h. Real-time quantitative PCR and immunoblotting analysis were carried out to monitor the RNA and protein expression levels of ITCH and Gli1. As shown in **Figures 1A,B**, the relative protein and mRNA level of ITCH was markedly up-regulated after Li treatment. The Li-induced changes in ITCH protein and mRNA were correlated with a reciprocal down-regulation of Gli1 levels (**Figures 1C,D**). These data suggest that lithium treatment promotes the degradation of Gli1 protein through regulating the expression of its E3 ligase ITCH level.

**FIGURE 2:**
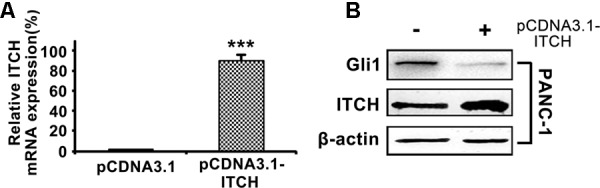
ITCH down-regulates the Gli1 level in PANC-1 cells. **(A)** The relative ITCH mRNA level in PANC-1 cells transfected with pCDNA3.1 or pCDNA3.1-ITCH. **(B)** The protein level of Gli1 and ITCH in PANC-1 cells after over-expressed with pCDNA3.1-ITCH. ^∗∗∗^*P* < 0.005.

To validate the regulatory function of ITCH on Gli1 in PDA, we over-expressed full-length ITCH in PANC-1 cells, and then measured Gli1 expression levels. Over-expression of ITCH (**Figure 2A**) markedly reduced Gli1 protein level (**Figure 2B**). These results confirmed that ITCH down-regulated Gli1 in PANC-1 cells.

### Lithium Promotes ITCH-Mediated Ubiquitination and Degradation of Gli1

**FIGURE 3:**
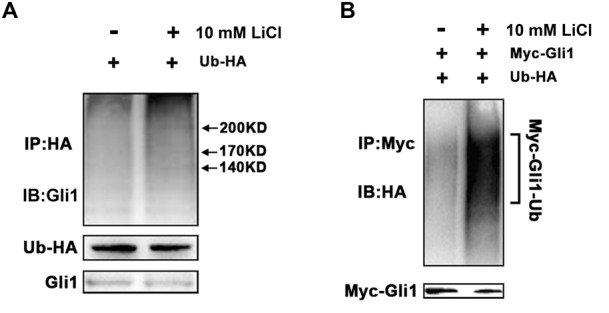
ITCH promotes ubiquitination and degradation of Gli1. **(A)** *In vitro* ubiquitination of Gli1 protein in the absent or present of Li treatment. PANC-1 cells were transfected with expression vectors encoding HA-ubiquitin and Myc-Gli1. More Gli1-HA-ubiquitin conjugates were detected when PANC-1 cells was treated by Li. **(B)** HA-ubiquitin and myc-Gli1 were co-expressed in PANC-1 cells, and more exogenous Myc-Gli1-HA-ubiquitin conjugates were detected with lithium treatment.

To determine if Li-mediated increase in cellular ITCH is associated with enhanced Gli1 ubiquitination, we firstly checked the rescue of Gli1 by inhibiting the proteasomal machinery. Increased protein level of Gli1 in PANC-1 cells were detected after treated with 2 μM MG132 only or co-treated with 2 μM MG132 and 10 mM LiCl (**Supplementary Figure S1**). Then we measured the Gli1 ubiquitination levels by *in vitro* ubiquitination assay, which showed that ITCH indeed facilitated the formation of ubiquitinated Gli1 in PANC-1 cells after the treatment of 10 mM LiCl (**Figure 3A**). Similar increased ubiquitylation were observed when PANC-1 cells were ectopically expressed with Myc-tagged Gli1 (**Figure 3B**). Taken together, these observations suggested that LiCl accelerated degradation of Gli1 through ITCH-mediated ubiquitylation.

**FIGURE 4:**
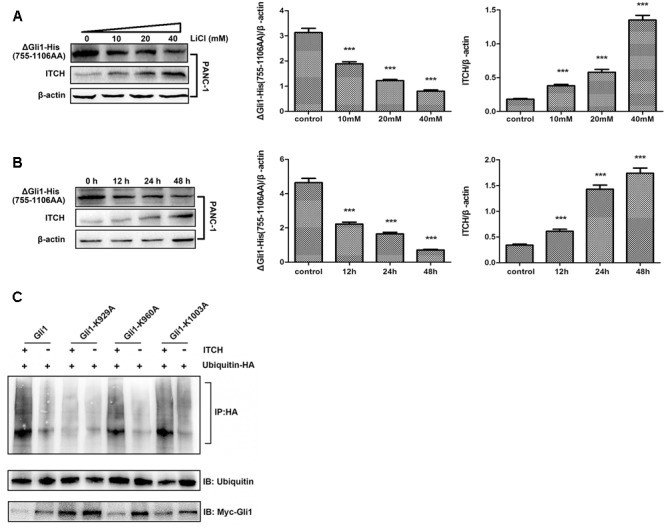
The identification of ubiquitylation sites in the C-terminus of Gli1. **(A)** The protein level of ΔGli1-His (755–1106 AA) was down-regulated when treated PANC-1 cells by 10, 20, or 40 mM lithium for 24 h. Histogram represented summarized results from three independent experiments. **(B)** The protein level of ΔGli1-His (755–1106 AA) was down-regulated when using 10 mM lithium to treated PANC-1 cells for 12, 24, or 48 h. Histogram represented summarized results from three independent experiments. **(C)** Three Gli1 mutants showed different levels of polyubiquitination, and K929A mutant was proved to be the key ubiquitylation site of Gli1 protein. ^∗∗∗^*P* < 0.005.

**FIGURE 5:**
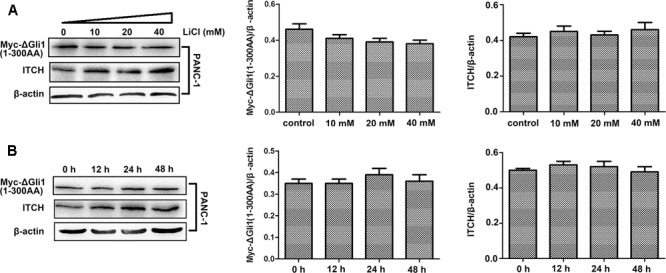
The identification of ubiquitylation sites in the N-terminus of Gli1. **(A)** The protein level of myc-ΔGli1 (1–300 AA) was not obviously changed when PANC-1 cells treated by 10, 20, or 40 mM lithium for 24 h. Histogram represented summarized results from three independent experiments. **(B)** The protein level of myc-ΔGli1 (1–300 AA) was not obviously changed when using 10 mM lithium treating PANC-1 cells for 12, 24, or 48 h. Histogram represented summarized results from three independent experiments.

Two major degrons, located at the N- or C-terminus of Gli1, respectively, have been identified to be important for Gli1 protein stability (Di Marcotullio et al., 2011). These observations, coupled with the fact that major predicted ubiquitination sites are clustered either at the N- or C-terminus, prompted us to determine which region might be important for Li/ITCH mediated Gli1 degradation. When deletion Gli1 construct, Myc-ΔGli1 (1–300 AA) or ΔGli1-His (755–1106 AA), was ectopically expressed in PANC-1 cells and treated with LiCl, the change in protein levels in response to Li treatment were different for the N- and C-terminal fragment Gli1. While the protein level of ΔGli1-His (755–1106 AA) was clearly down-regulated dose-dependently in response to LiCl treatment (**Figures 4A,B**), no obvious change of Myc-ΔGli1 (1–300 AA) protein level was detected after LiCl treatment (**Figure 5**). These results suggest that the C-terminal region between residues 755 and 1106 of Gli1 is important for Li/ITCH induced Gli1 degradation.

To further map the potential ITCH related ubiquitination site in Gli1, we individually mutated the potential ubiquitination sites at the C-terminal of Gli1, K929, K960, and K1003. *In vitro* ubiquitination assay of full-length WT Gli1, Gli1-K929A, Gli1-K960A, and Gli1-K1003A showed that while mutation of K929 effectively blocked Gli1 ubiquitination, Gli1-K960A and Gli1-K1003A underwent robust ubiquitination as the WT Gli1 when co-transfected with ubiquitin-HA. In addition, the cellular expression levels of the Gli1 were inversely correlated with the levels of ubiquitination with significant accumulation of the Gli1-K929A as compared to WT Gli, Gli1-K960A and Gli1-K1003A (**Figure 4C**). These results demonstrated that K929 is the major ubiquitination site in Gli1 responsible for Gli1 protein stability in PANC-1 cells.

### Lithium Inhibits GSK3β and Stabilizes SUFU/Gli1 Complex

**FIGURE 6:**
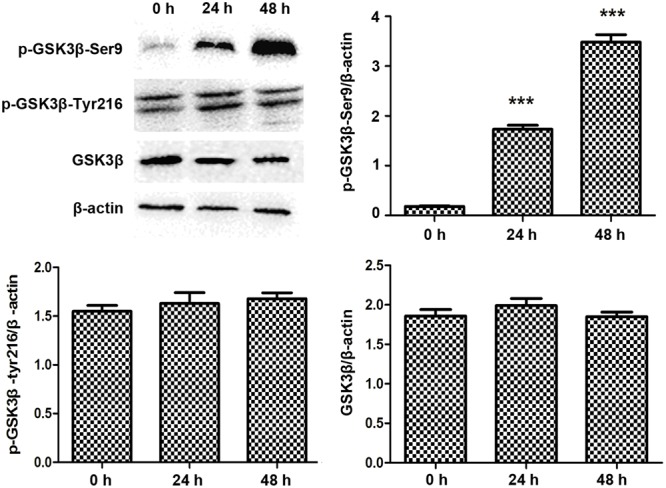
Lithium increases GSK3β Ser9 phosphorylation. Immunoblotting of Ser9 phosphorylated GSK3β showed an increase after 10 mM lithium treatment compared with total GSK3β and Tyr216 phosphorylated GSK3β, which remained constant. Histogram represented summarized results of p-GSK3β-Ser9, p-GSK3β-Tyr216, and GSK3β from three independent experiments. ^∗∗∗^*P* < 0.005.

Lithium is known for promoting the inhibitory Ser9 phophorylation of GSK3β (Jope, 2003), so we checked the Ser9 phophorylation status of GSK3β in PANC-1 cells before and after treatment with LiCl. While total and Tyr216 phophorylated GSK3β levels remained constant regardless of the treatments, the phophorylated Ser9 fraction of GSK3β increased after treatment with LiCl (**Figure 6**).

**FIGURE 7:**
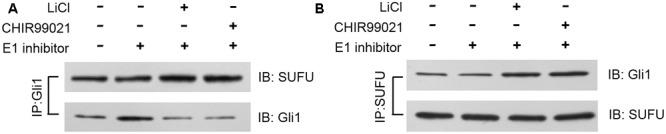
Lithium enhances Gli1-SUFU complex formation. Immunoblotting images of co-immunoprecipitation experiments, showing increased binding of Gli1 and SUFU after 10 mM lithium or 10 μM CHIR99021 treatment for 24 h in the presence of 2 μM E1 inhibitor PYR-41. **(A)** Using anti-Gli1 antibody to immunoprecipitation Gli1/SUFU complex. **(B)** Using anti-SUFU antibody to immunoprecipitation Gli1/SUFU complex showed increased Gli1 level.

**FIGURE 8:**
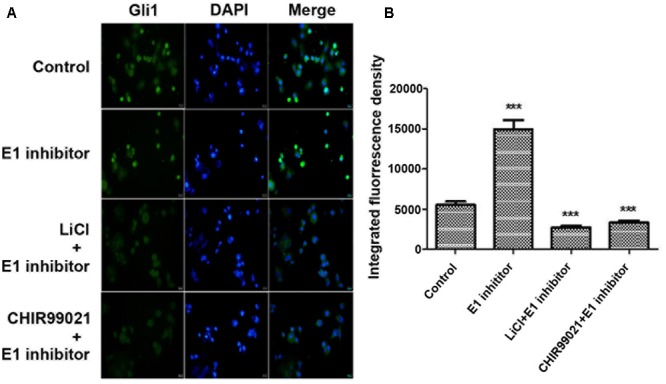
Lithium reduces Gli11 level in nucleus. **(A)** Immunofluorescence staining showing a decreased Gli1 level in nucleus of PANC-1 cells after 10 mM lithium or 10 μM CHIR99021 treatment for 24 h in the presence of 2 μM E1 inhibitor PYR-41. **(B)** Integrated fluorescence density of Gli1 in nucleus analyzed by Image J software. ^∗∗∗^*P* < 0.005.

SUFU is a negative Hh regulator that binds and sequesters Gli1 in its inactive state in the cytosol. It has been reported that GSK3β can binds and phosphorylates SUFU and destabilizes its interaction with Gli1, which allows the dissociation and subsequent translocation of Gli1 into nucleus where Gli1 activates the transcription of Hh target genes (Takenaka et al., 2007). To check if lithium treatment can lead to increased cellular levels of SUFU/Gli1 complex, we performed affinity immunoprecipitation experiment to probe the effect of lithium treatment on cellular interaction between SUFU and Gli1. As shown in **Figure 7A**, following LiCl treatment, the amount of SUFU associated with immunoprecipitation that was pulled down using an anti-Gli1 antibody increased despite a reduced the total cellular Gli1 level. Conversely the level of Gli1 associated with immunoprecipitation from pulldown experiment performed using anti-SUFU antibody was also increased as expected (**Figure 7B**). These observations suggest that lithium treatment stabilizes SUFU/Gli1 complex, which sequesters Gli1 in cytosol. Consistent with this notion, the nuclear/cytosol ratio of Gli1 was significantly decreased in lithium-treated cells (**Figure 8**). Similar effects of CHIR99021 on SUFU-Gli1 interaction and Gli1 nuclear localization were also detected (**Figures 7, 8**).

## Discussion

The Hh signaling is a key pathway important for animal development and dysregulation of Hh pathway has been implicated in major human diseases including cancer. As a major down-stream effector of Hh signaling, the expression level and transcriptional activity of Gli1 is tightly regulated to maintain proper homeostasis. Ubiquitination-mediated protein degradation of Gli1 represents one major mechanism of Gli1 regulation. To date, it has been reported that Gli1 can be targeted by a member of E3 ubiquitin ligases, including β-TrCP and ITCH (Di Marcotullio et al., 2007). Phosphorylation of Gli protein by GSK3β, as well as PKA and CKI, has been shown to recruit β-TrCP, resulting in polyubiquitination and eventual proteasome-dependent degradation of Gli1. The degradation signals important for β-TrCP mediated regulation are located in the N-terminal half of Gli1 (Huntzicker et al., 2006). On the other hand, HECT family E3 ubiquitin ligase ITCH binds with Gli1 at its C-terminal and facilities its poly-ubiquitylation (Di Marcotullio et al., 2011). Since our previous study demonstrates that lithium suppresses GSK3β activity and destabilizes Gli1 protein in PDA cells, it unlikely that lithium’s effect is mediated by β-TrCP as inhibition of GSK3β should results in less β-TrCP recruitment and reduced Gli1 degradation. Our results also showed no changes of the protein levels of β-TrCP and PCAF in PANC-1 cells after lithium treatment (**Supplementary Figure S2**). We therefore focused on our efforts on the HECT-type E3 ubiquitin ligase-dependent pathway. Our study indeed reveals that lithium upregulates ITCH, promotes polyubiquitination and degradation of Gli1. Through deletion and site-specific mutagenesis analysis, coupled with *in vitro* ubiquitination assay, we find that K929 is the key ubiquitination site targeted by lithium/ITCH in Gli1 protein. This is an important finding as while ITCH-mediated Gli1 ubiquitination was initially proposed in 2006 the precise ITCH ubiquitination site in Gli1 has not been identified.

In addition to be regulated by ubiquitination, Gli1 can also be modulated by SUFU, which interacts with and inhibits Gli1 activation by sequestering it in the cytosol (Ding et al., 1999; Kogerman et al., 1999; Stone et al., 1999). Interaction between SUFU and Gli1 can be disrupted by GSK3β, which phosphorylates SUFU. While conventionally, GSK3β is considered as a negative regulator of Hh signaling pathway, by promoting the phosphorylation of Gli1 protein, subsequently its ubiquitination and degradation. However, in PDA high expression levels of GSK3β (Ougolkov et al., 2006) coexist with the aberrantly activation in Hh signaling pathway (Thayer et al., 2003; Peng et al., 2013), suggesting that GSK3β may play a complicated role in PDA cells. Indeed, our results show that lithium inhibits GSK3β and enhances interaction between SUFU and Gli1, which decreases Gli1 translocation to nucleus and therefore its transcriptional activity.

Hedgehog pathway inhibition has been proven to be an effective anti-cancer therapeutic strategy and the inhibitors or small molecular modulators for Hh/Gli signaling target on SMO and Gli1 (Rimkus et al., 2016). A gain-of-function isoform of the Gli1 transcription factor with 41 amino acids deleted near the N-terminus was identified as tGli1 (Lo et al., 2009; Carpenter and Lo, 2012). The exclusive expression of tGli1 in tumor tissues and its unique role in tumorigenesis make it a potential target in anticancer therapies but by far no small molecular inhibitor has been developed against this shortened Gli1 subtype (Carpenter and Lo, 2012; Rimkus et al., 2016). Thus, ITCH mediated Gli1 ubiquitination/degradation induced by lithium may be one of effective degradation pathways for tGli1. GSK3β is a critical therapeutic target in canonical Wnt/β-catenin and PI3K/Akt signaling pathway, and several known GSK3β inhibitors also take part in over activated Hh signaling in PDA (Pandey and DeGrado, 2016). The inhibition of GSK3β-mediated SUFU-Gli1 destabilization can be a new pharmacological function of GSK3β inhibitors that are effective in Hh signaling suppression. Moreover, it has been shown that lithium induces apoptosis of a variety of cancer cells and synergistically enhances the anti-cancer effect of gemcitabine (Schotte et al., 2001; Kaufmann et al., 2011; Peng et al., 2013). All these findings of Lithium’s function provide a potentially new therapeutic strategy for PDA through targeting Gli1.

In summary, our study reveals a novel mechanism by which lithium suppresses Hh/Gli1 signaling in PDA by promoting ITCH mediated Gli1 ubiquitination/degradation and by suppressing GSK3β-mediated SUFU-Gli1 dissociation, thus Gli1 nuclear translocation.

## Author Contributions

Conceived and designed the experiments: XW, XC, and ML. Performed the experiments: XW, ZF, and ML. Analyzed the data: XW, ZF, AW, CL, XC, and ML. Contributed reagents/materials/analysis tools: XC and ML. Wrote the manuscript: XW, ZF, XC, and ML. All authors have read and approved the final manuscript.

## Conflict of Interest Statement

The authors declare that the research was conducted in the absence of any commercial or financial relationships that could be construed as a potential conflict of interest.

## Supplementary Material

FIGURE S1 MG132 treatment retards the proteasome-mediated degradation of Gli1. 2 μM MG132 treatment or co-treatment with 2 μM MG132 and 10 mM LiCl increased protein level of Gli1 in PANC-1 cells. Histogram represented summarized results from three independent experiments. ^∗∗∗^*P* < 0.005.

FIGURE S2 Lithium treatment does not affect the protein level of β-TrCP and PCAF. Representative images of β-TrCP and PCAF after the treatment of 10 mM lithium for 24 h. Histogram represented summarized results from three independent experiments.
